# Knowledge Engineering in Chemistry: From Expert Systems to Agents of Creation

**DOI:** 10.1021/acs.accounts.2c00617

**Published:** 2022-12-14

**Authors:** Aleksandar Kondinski, Jiaru Bai, Sebastian Mosbach, Jethro Akroyd, Markus Kraft

**Affiliations:** † Department of Chemical Engineering and Biotechnology, University of Cambridge, Philippa Fawcett Drive, Cambridge CB3 0AS, U.K.; ‡ CARES, Cambridge Centre for Advanced Research and Education in Singapore, 1 Create Way, CREATE Tower, #05-05, 138602 Singapore; ¶ CMCL Innovations, Sheraton House, Castle Park, Cambridge CB3 0AX, U.K.; § School of Chemical and Biomedical Engineering, Nanyang Technological University, 62 Nanyang Drive, 637459 Singapore

## Abstract

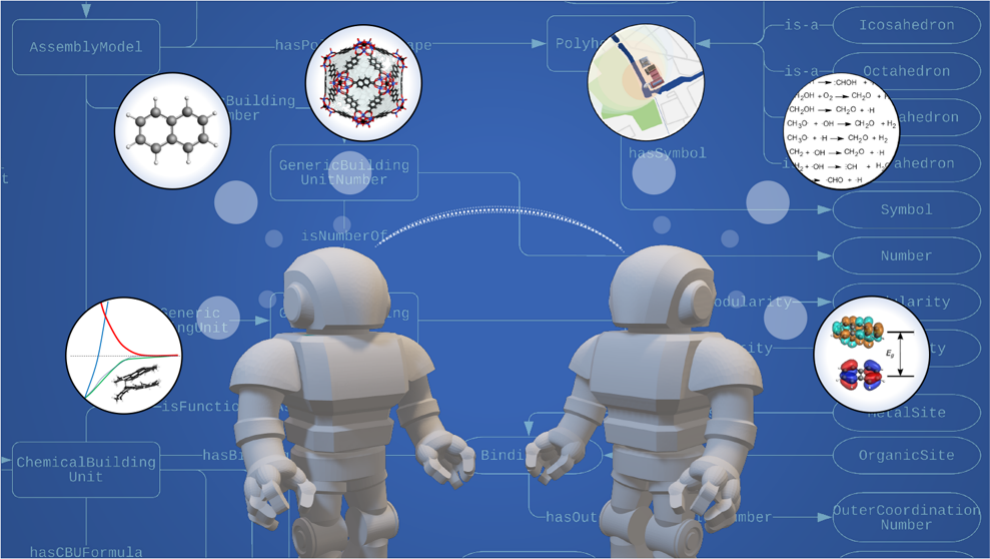

Passing knowledge from human to human is a natural process that has continued since the beginning of humankind. Over the past few decades, we have witnessed that knowledge is no longer passed only between humans but also from humans to machines. The latter form of knowledge transfer represents a cornerstone in artificial intelligence (AI) and lays the foundation for knowledge engineering (KE). In order to pass knowledge to machines, humans need to structure, formalize, and make knowledge machine-readable. Subsequently, humans also need to develop software that emulates their decision-making process. In order to engineer chemical knowledge, chemists are often required to challenge their understanding of chemistry and thinking processes, which may help improve the structure of chemical knowledge.

Knowledge engineering in chemistry dates from the development of expert systems that emulated the thinking process of analytical and organic chemists. Since then, many different expert systems employing rather limited knowledge bases have been developed, solving problems in retrosynthesis, analytical chemistry, chemical risk assessment, etc. However, toward the end of the 20th century, the AI winters slowed down the development of expert systems for chemistry. At the same time, the increasing complexity of chemical research, alongside the limitations of the available computing tools, made it difficult for many chemistry expert systems to keep pace.

In the past two decades, the semantic web, the popularization of object-oriented programming, and the increase in computational power have revitalized knowledge engineering. Knowledge formalization through ontologies has become commonplace, triggering the subsequent development of knowledge graphs and cognitive software agents. These tools enable the possibility of interoperability, enabling the representation of more complex systems, inference capabilities, and the synthesis of new knowledge.

This Account introduces the history, the core principles of KE, and its applications within the broad realm of chemical research and engineering. In this regard, we first discuss how chemical knowledge is formalized and how a chemist’s cognition can be emulated with the help of reasoning algorithms. Following this, we discuss various applications of knowledge graph and agent technology used to solve problems in chemistry related to molecular engineering, chemical mechanisms, multiscale modeling, automation of calculations and experiments, and chemist–machine interactions. These developments are discussed in the context of a universal and dynamic knowledge ecosystem, referred to as The World Avatar (TWA).

## Key References

- KondinskiA.; MenonA.; NurkowskiD.; FaraziF.; MosbachS.; AkroydJ.; KraftM.Automated Rational Design of Metal-Organic Polyhedra. J. Am. Chem. Soc.2022, 144, 11713–117283573195410.1021/jacs.2c03402PMC9264355.^1^ A knowledge graph and an agent emulate inductive reasoning and rationally derive the construction of new materials.
- MosbachS.; MenonA.; FaraziF.; KrdzavacN.; ZhouX.; AkroydJ.; KraftM.Multiscale cross-domain thermochemical knowledge-graph. J. Chem. Inf. Model2020, 60, 6155–61663324224310.1021/acs.jcim.0c01145.^2^ Agents use chemical data from the knowledge graph, real-time data, and dispersion models to investigate emissions to the environment.
- FaraziF.; AkroydJ.; MosbachS.; BuergerP.; NurkowskiD.; SalamancaM.; KraftM.OntoKin: An ontology for chemical kinetic reaction mechanisms. J. Chem. Inf. Model2020, 60, 108–1203184632310.1021/acs.jcim.9b00960.^3^ The OntoKin ontology served as one of the early blueprints guiding the ontological representation of chemical knowledge.
- ZhouX.; NurkowskiD.; MosbachS.; AkroydJ.; KraftM.Question answering system for chemistry. J. Chem. Inf. Model2021, 61, 3868–38803433850410.1021/acs.jcim.1c00275.^4^ Agents, knowledge graph, and natural language processing are synergistically combined to provide chemists with friendly access to the world of chemical information.

## 1 Introduction

**Figure 1:**
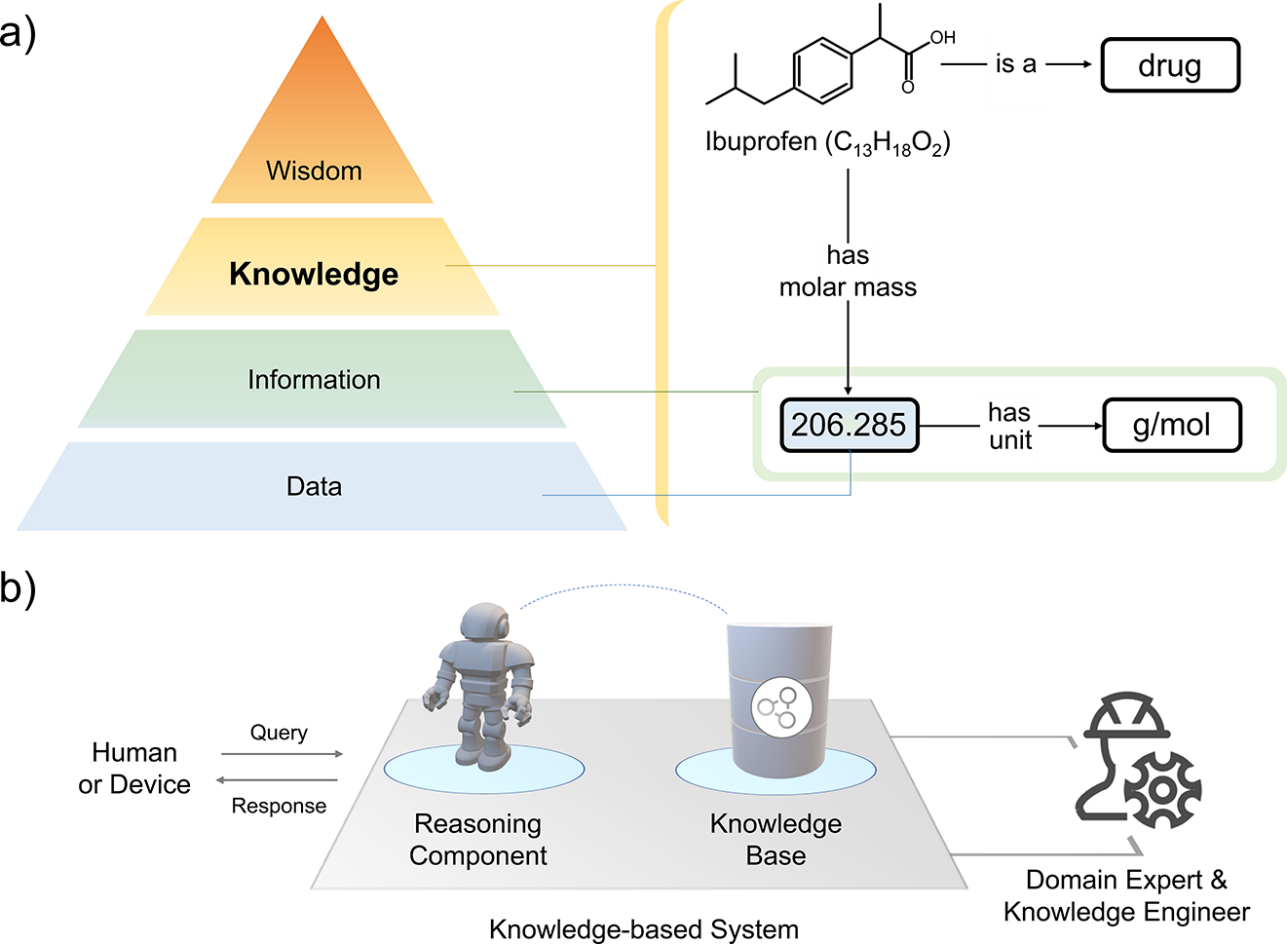
(a) Schematic representation of the “DIKW pyramid” illustrating the meaning of data, information, and knowledge in the chemical context. (b) The minimal components of a knowledge-based system.

Knowledge is the focal subject of philosophical disciplines such as epistemology and metaphysics. When viewed from the perspective of information science, knowledge is described hierarchically and relative to data, information, and wisdom (see Figure 1a).^5^ The “DIKW” pyramid places data at the bottom of the hierarchy; thus, a data point such as “206.285” can exist without the necessity of having a meaning. A data point that is given relation can become meaningful and thus described as a piece of information. “206.285 g/mol” is a form of information that likely refers to some form of a molar mass. The collection of information in a way that becomes useful is regarded as knowledge. Thus, “ibuprofen” is a “drug” with formula “C_16_H_18_O_12_”, and molar mass of “206.285 g/mol” would be a form of (minimal) knowledge. Making reasoned and educated judgments or decisions based on knowledge is the basis of wisdom.^5^

Knowledge of chemical processes has been documented since antiquity. However, most of this knowledge throughout most of time in human history has remained esoteric, poorly understood, and shared among a tiny minority of people.^6^ The scientific revolution introduced reasoned structuring of knowledge, which in chemistry was followed by the adoption of common chemical representations (e.g., symbols, equations, structures) and standards in reporting new chemistry, thus making the subject more widely understandable.^6^ Following the second World War, a number of visionary ideas such as the Turing test,^7^ general problem solvers,^8^ and universal constructors^9^ appeared, that laid the foundations of artificial intelligence (AI), whose further sophistication was realized to depend not only on computing but also on advances in the understanding of human cognition.^10^

Knowledge Engineering (KE) is one of the first and most successful branches of AI that emerged in the 1960s. The original aim of KE is to emulate the decision-making process of a human expert,^11^ consequently leading to the development of many commercialized expert and knowledge management systems, commonly referred to as knowledge-based systems.^11,12^ A knowledge-based system is a fundamentally constructed knowledge base which documents knowledge in a machine-readable way and a reasoning component (i.e., an inference engine) that, following a request from a user, queries the knowledge base and provides reasoned answers. The knowledge base is commonly maintained by people with domain and knowledge engineering expertise (see Figure 1b).

In the early days of KE, chemistry formulated problems that KE systems could address and demonstrate potential (e.g., meaningful hypothesis generation^13^). However, the AI winters in the 20th century and the general disinterest of the chemistry community in AI systems disrupted the continued development of such systems for chemistry research.^14^ However, more recent technological advances (e.g., inexpensive computational power, free software, popularization of programming) reopened interest in this field. A major game-changer was the conceptualization of the Semantic Web by Tim Berners-Lee in the late 1990s/early 2000s,^15^ which gradually transformed into a knowledge graph (KG) approach.^16,17^ KGs based on the Semantic Web can interlink heterogeneous data and make it accessible to (autonomous) software agents.^11^ In addition to querying KGs, these agents were conceptualized as performing different tasks that involve reasoning, learning from humans, and operating on infrastructure to create new things (e.g., knowledge, services, and physical items, including chemicals). Owing to these qualities, agents have been respectively referred to as “intelligent agents”,^15^ “disciple agents”,^11^ and “agents of creation”.^18^

In this work, we first introduce the conceptual basics of KE: the formalization of chemical knowledge and reasoning and the route to knowledge systems engineering. We then discuss the beginning of KE in chemistry through examples of legacy expert systems and proceed with the current implementations of a knowledge ecosystem where chemistry plays a central role. The latter technological implementation is illustrated with many examples of navigation through reaction complexity, multiscale modeling, rational design of self-assembled materials, and friendly interactions with chemical KG. Lastly, we outline existing challenges in capturing knowledge dynamics and provide a perspective for future developments.

## 2 Chemical Knowledge and Reasoning

How do KE systems emulate expert-like decision-making? To answer this question, we first look into the meaning of chemical knowledge formalization and the navigation through knowledge based on reasoning. Then we outline the main stages of KE project development.

### 2.1 Formal Representation of Chemical Knowledge

**Figure 2:**
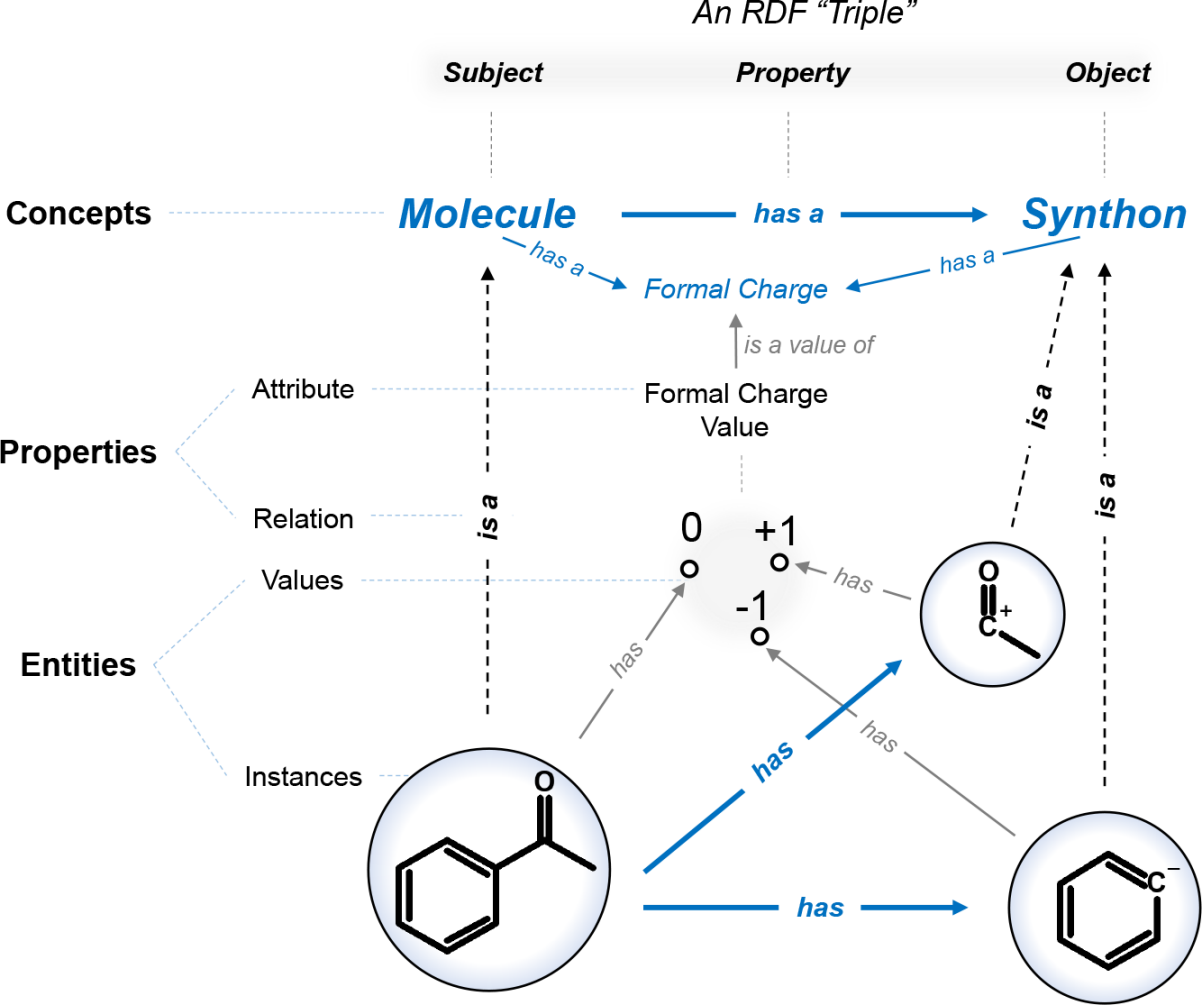
Mapping the relationship between molecule (chemical) and synthon (abstract) concepts and illustrating them with instances. Description of an RDF triple (top) and ontology stacking (left).

In order to map knowledge, a machine needs to ascribe meaning to data and find a relationship between data points. Documenting data in a relational format, that is, through many interconnected tables, is a straightforward but very restrictive format when it comes to changes in the knowledge structure.^19^ Knowledge graphs (KGs) are a different approach where a data point can act as a node that links to other entities in the graph via well-defined relationships. New relations and data can be added to the KG without disturbing the preexisting knowledge structure. Structured data consistency in the framework of KGs is achieved using blueprint networks (i.e., schema) that describe how different concepts and properties link to one another. These forms of schemas are commonly referred to as ontologies, defining the terminological box (TBox) of a KG. Knowledge instantiated based on an ontology represents the assertion component (ABox), and it is used in the actual population of the KG. As an ontology, like any directed graph, can be represented as a collection of “triples”, that is, subject-predicate-object statements, a database hosting (a part of) the KG is commonly referred to as a triple store. Although knowledge systems can solve real-world problems, many concepts they embody may vary in abstraction. A concept such as “chemical compound” has physical existence; however, “synthon” is a concept that refers to the mental imagery of a compound fragment. In other words, a synthon is not something one can purchase (see Figure 2). When using Semantic Web technology, all concepts and data are linked via unique Internationalized Resource Identifiers (IRIs), making them unambiguously identifiable.^15^ In chemistry, the Chemical Entities of Biological Interest (ChEBI) is one of the deepest ontologies with database implementation focusing on small chemical compounds.^20,21^

### 2.2 Evidence-Based Reasoning

**Figure 3:**
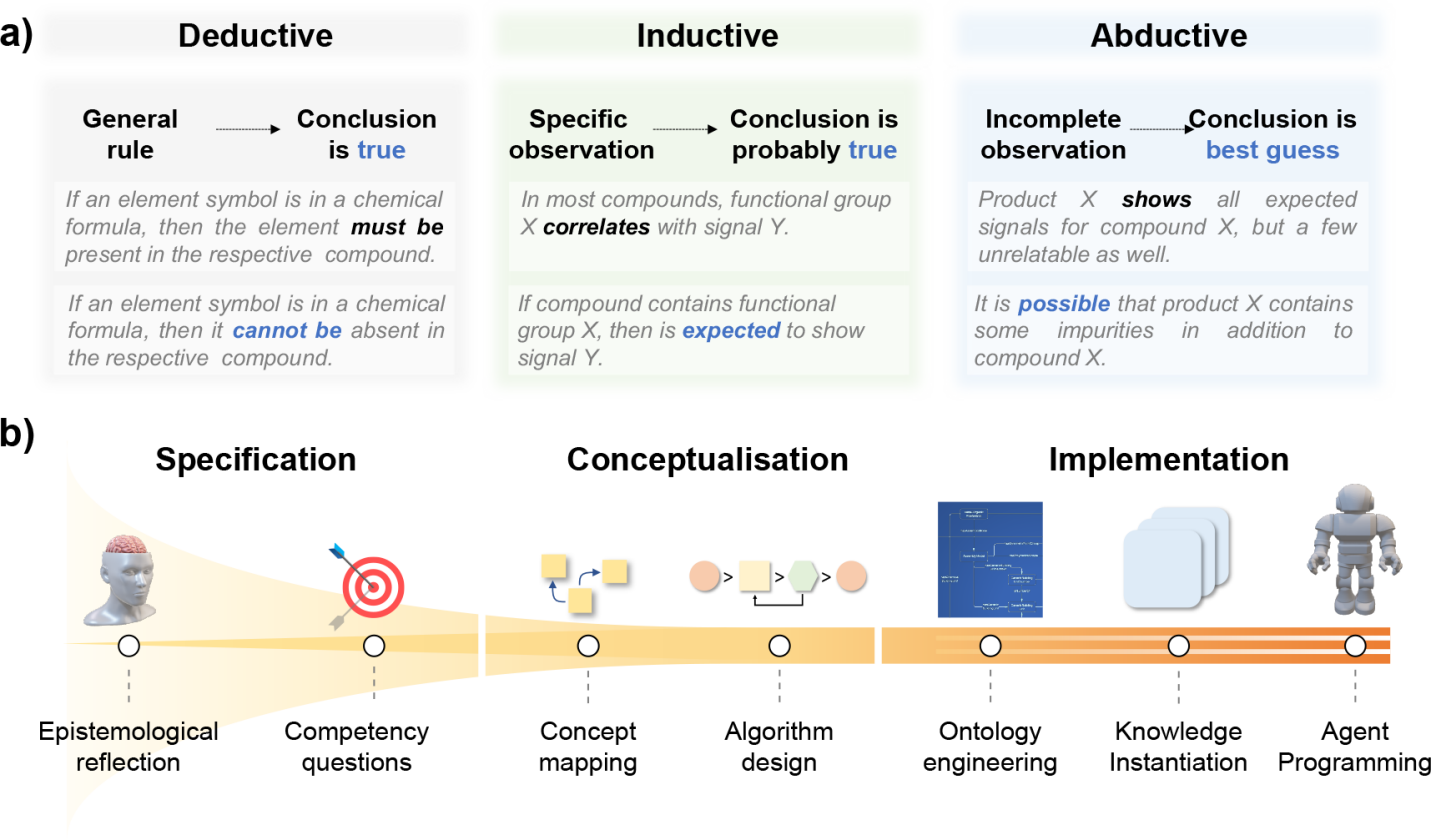
(a) The three main types of reasoning, illustrated with general case scenarios in chemistry. (b) The three main stages in KE project development.

Humans typically apply three forms of reasoning,^11^ such as (i) based on logic and fixed premises (i.e., deductive); (ii) derived from statistical or anecdotal reference (i.e., inductive); (iii) based on imagination and best guess (i.e., abductive) (see Figure 3a). Abduction remains broadly accepted as the most challenging to be implemented in AI systems. The different forms of reasoning often manifest themselves in human cognition through mental shortcuts called heuristics.^22^ When heuristics are used as part of programming, their utility is primarily to reveal a viable solution by disregarding unlikely solutions. In many expert systems, heuristics have been implemented as deductive reasoners (i.e., rules). In our view, this may not be the best practice as it blurs the line between a rule (i.e., guaranteed outcome) and a likely (i.e., not entirely certain) outcome. Consequently, “rules”, especially those in the context of retrosynthetic analysis,^23^ risk becoming criticized for any possible shortcoming of an expert system implementation.

Over the past decade, machine learning (ML) has increased its dominance in extracting intelligence from chemical data.^24^ However, this technique has been particularly successful in domains where clean data is plentiful.^25^ ML makes inferences based on associations deriving from data; in principle, ML does not need knowledge or understanding of behavior to make those associations. As associations are based on statistical significance, ML may also be viewed as a practical implementation of inductive reasoning.^26^ On the other hand, KE is developed based on the knowledge and experiences of a domain expert. Thus, algorithms in KE do not need to be pretrained with data, which is a way forward for cases where data is scarce. Our recent work in metal–organic polyhedra (MOPs) vividly illustrates this as the key algorithm embodies inductive reasoning through set operations, effectively deriving new and rational self-assemblies.^1^

Despite the differences, both deductive and inductive reasoning are deeply dependent on the presence of available unambiguous evidence. Working through cases where there is ambiguity (e.g., contradictory reports on the same event/thing) cognitively may not differ from cases where evidence is incomplete. Such scenarios require a higher level of abstraction and fall in the realms of abductive reasoning. Depending on the domain and the case complexity, they may be cognitively very challenging even for the human expert.

### 2.3 Stages in KE Project Development

A KE project starts with a genuine problem that a person or a team would like to solve and undergoes three general stages: specification, conceptualization, and implementation (see Figure 3b).^27^ In the specification stage, the experts do what we would refer to as “epistemological reflection”, formulating what they know and how they know it. The team then defines a list of competency questions that the desired KE system is meant to realistically solve. These two aspects effectively narrow down the main focus and goal of the KE system, and they lay the foundations for the conceptualization stage where concept maps are first formulated.^28^ A concept map enables a semiformal representation of knowledge and provides preliminary insight into the type and number of involved entities. Experts may define or design an algorithm suitable for making inferences and tackling one or more competency questions in conjunction with the concept map.^29^

During the implementation stage, the entities of the concept map are ontologized. Experts clean information and instantiate knowledge based on the ontological format. This completes the assertion component that populates the KG. Finally, based on the designed algorithm, an agent capable of traversing the KG and making inferences is programmed. The overall system is then tested and placed in use. Multiple iterations across the three stages are not uncommon, and they often contribute toward better project outcomes.^29^

## 3 Legacy Expert Systems

**Figure 4:**
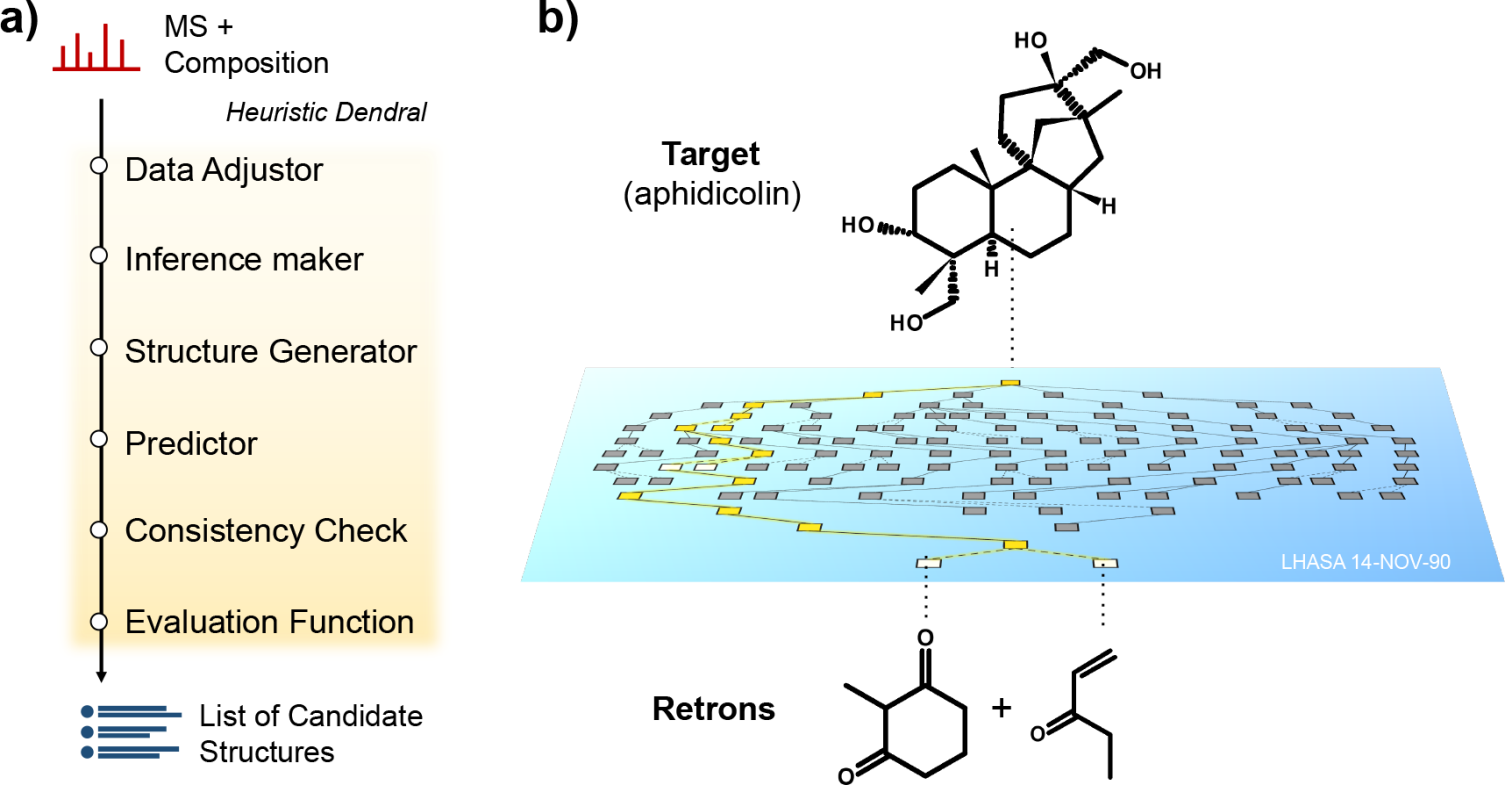
(a) The workflow of Heuristic Dendral. (b) Deriving a retrosynthetic pathway to aphidicolin (an antibiotic) using LHASA based on Corey’s talk in his Nobel Lecture.

During the 1960s, two major legacy expert systems essentially pioneered KE. The Dendral project started in 1965 and was developed in the context of NASA’s Mars exploration, where real-time molecule detection and elucidation systems were needed. This inspired a group of leading scientists at Stanford University, such as Carl Djerassi, Edward Feigenbaum, and Joshua Lederberg, to automate mass spectrometric species elucidation.^30^ Regarding software architecture, Dendral was subdivided into Heuristic Dendral, a component that elucidates species, and Metadendral, a component that learns new rules on how species are fragmented.^31^ The two components were envisioned to work in a way that ensures continuous learning. For the development of the Heuristic Dendral, the team developed a general workflow, integrating multiple algorithms for combinatorial exploration of the chemical space and a knowledge base of mass spectrometry fragmentation rules (see Figure 4a). However, the development of Metadendral has remained challenging. One reason may be that the team attempted to tackle the problem of dynamic knowledge before practical implementation on how to achieve that could be possible.

In 1967, Elias Corey (Harvard University) conceptualized and structured retrosynthesis in the form of five general strategies.^32^ In 1969, Corey and Wipke developed the first organic synthesis planning expert system^33^ that later became better known as “Logic and Heuristics Applied to Synthetic Analysis” (LHASA).^34^ Over the past decades, LHASA boasted several design strategies and encoded group-protection information, and generally, it served as a blueprint for how to build retrosynthetic expert systems.^23^ In 1990, Corey was awarded the Nobel Prize in Chemistry “for his development of the theory and methodology of organic synthesis”, with the developments and usage of LHASA playing an essential role in his Nobel Lecture (see Figure 4b).^35^

These legacy expert systems in chemistry were followed by many other examples, beautifully discussed and illustrated in the books of Judson^14^ and Hemmer.^12^ The expert systems also placed a technical necessity for finding efficient ways to store and share chemical information, which consequently laid a genuine purpose for developing cheminformatics.^36^ Although not broadly acknowledged, some scientists, such as Corey himself, also appreciated the value of KE beyond its implementation. On a deeper level, KE requires chemists to think more generally about their subject and occasionally find more efficient ways to structure chemical knowledge.^14,34^

## 4 The World Avatar: A Universal World Model

**Figure 5:**
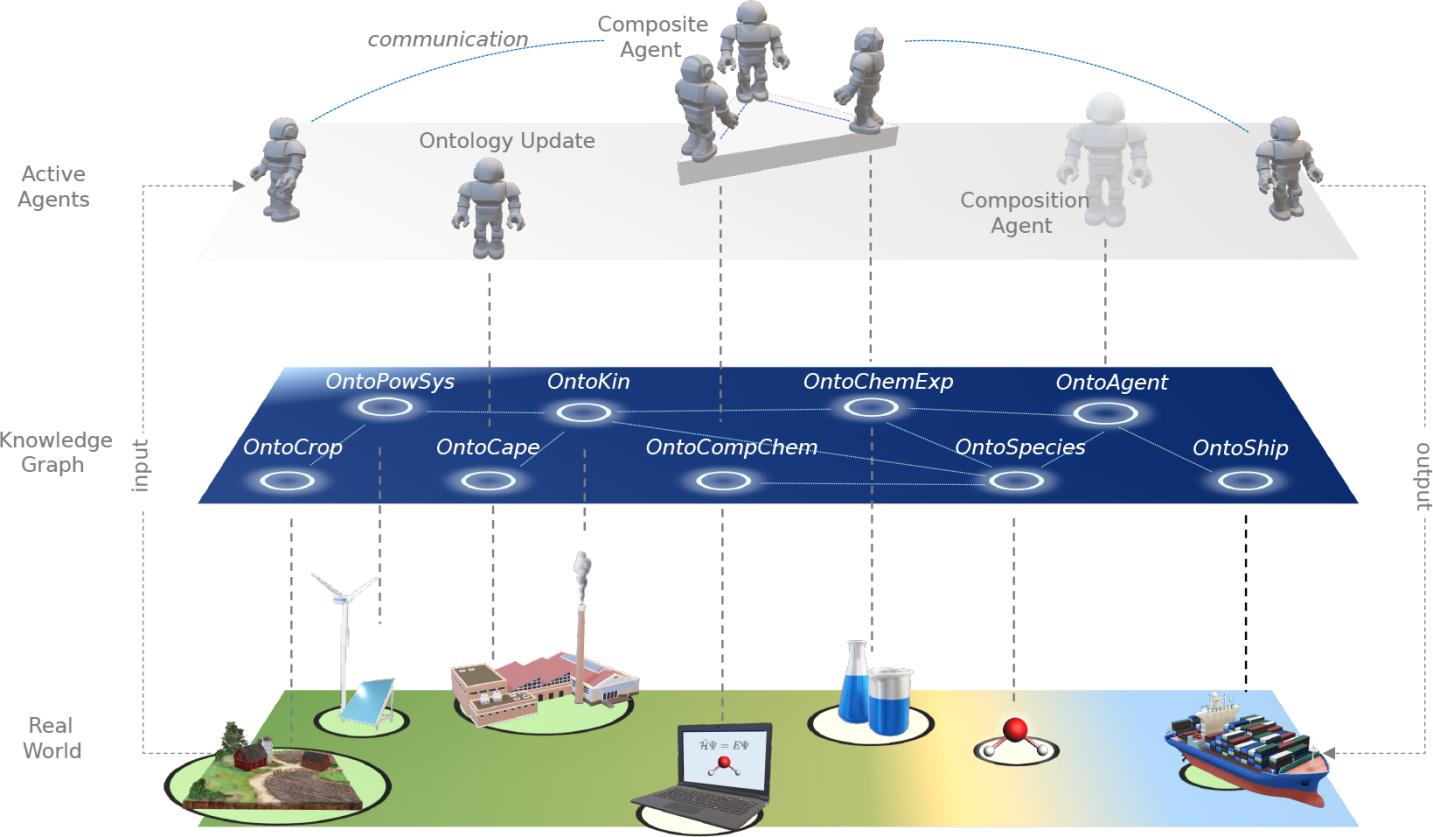
Three layers of TWA (www.theworldavatar.com) digital twin of the real world.

Not long after conceptualizing the Semantic Web,^15^ leading cheminformatics researchers realized how beneficial this technology could be to chemists.^37,38^ However, how we can make the broader community benefit from the semantic instantiation of chemistry was envisioned by us in 2010.^39^ In this regard, we outlined the necessity for semantic instantiating of the chemical industry complex and the environmental impact from combustion as two very relevant subjects able to bridge molecular-scale chemistry to real-world macroscale phenomena with socioeconomic, environmental, and health impacts. Our early vision was practically implemented as part of our effort to digitalize ecofriendly chemical industry parks,^40,41^ such as the one located on Jurong Island (Singapore). The latter attempt initially led to the foundations of the “J-Park Simulator” (JPS).^40,41^ JPS embodied many aspects beyond chemical engineering affecting productivity and environment, such as logistics, infrastructure, energy usage, and waste, among others.^42−44^ By building digital tools to represent these aspects, it was realized that they are more widely applicable than just to chemical parks but more broadly to the world at large, leading to the extension and transition of the JPS to the ongoing “The World Avatar” project (TWA), an effort to create an all encompassing universal world model.^45,46^ Although TWA (see Figure 5) at first sight may appear as a bold and overambitious project, recently more leading figures in computer science and environmental studies have embraced the world-centric idea as a necessity for the progression of contemporary AI.^47,48^

Digital twins are an emerging technology that provides a real-time representation of real-world phenomena, assisting decision-making by exploration of what-if scenarios.^49^ In this regard, TWA (see Figure 5) has been conceptualized as a universal digital twin based on the Semantic Web, where a universal KG maps the real world. TWA follows the FAIR principles of linked data, that is, all stored knowledge is findable, accessible, interoperable, and reusable.^50^ On the “top” of the knowledge layer, TWA integrates a layer of active agents that operate on it.^44^ These agents differ from the classical inference engines employed in expert systems, and they have a number of different tasks such as (i) implementing information pipelines; (ii) sending signals back to the real world; (iii) providing an interface to computational models; (iv) restructuring the KG by adding instances, concepts, and relationships; and (v) discovering, combining, and composing new agents capable of performing new and on-demand tasks.^44,46^ At the same time, agents are also represented through concepts, instances, and properties in the knowledge graph. The latter feature makes agents findable and enables the possibility of solving complex tasks through interagent communication and collaboration.^44^

## 5 Chemistry as Part of a Knowledge Ecosystem

Currently, a number of high-tech companies, Google, IBM, Microsoft, Facebook, and eBay, have been implementing KGs on an industrial scale.^51^ In the context of the pharmaceutical industry, AstraZeneca is a company that openly leads the way on KGs as part of their drug discovery.^52^ This section discusses the development of a chemistry KG and related agents as part of TWA knowledge ecosystem.^43,45^

### 5.1 Chemical Species

**Figure 6:**
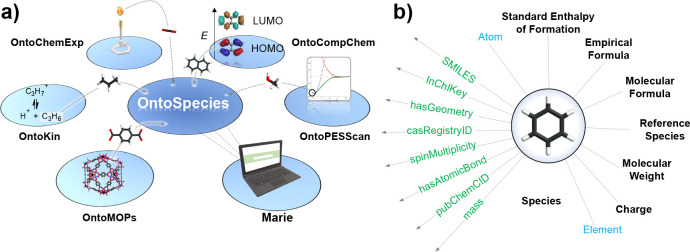
(a) Connection of OntoSpecies to other segments of TWA KG. (b) Key OntoSpecies (black) and external (blue) concepts, along with a number of properties (green) used to describe chemical species in TWA KG.

OntoSpecies is an ontology that describes unique chemical species and their chemical properties in TWA. In TWA, species are assigned IRIs, allowing their unique identification.^53^ OntoSpecies plays a central role, enabling the linking of species to instances and concepts deriving from other ontologies in TWA KG (see Figure 6a). A chemical species in OntoSpecies has a recorded molecular formula, charge, molecular weight, and spin multiplicity. Species that are based on different isotopes, charges, and spin states are treated as different chemical species. By assigning different IRIs to species which incorporate universally unique identifiers (UUIDs), OntoSpecies becomes relevant for the digital representation of isotope labeling experiments, redox and electrochemically driven processes, and photochemistry. Considering reactor simulations, OntoSpecies records standard enthalpy of formation along with its contextual information such as reference temperature, state, and provenance.^53^

As identification solely based on IRIs may be machine actionable but not directly meaningful to chemists, chemical species in TWA are further labeled with common cheminformatic identifiers,^54^ such as InChI, InChIKey, CAS registry number, PubChemCID, and SMILES (see Figure 6b). These labeling identifiers facilitate searching for additional information on external resources. OntoSpecies also records the molecular geometry of different species semantically, meaning that every bond and atom is uniquely identified with an IRI. The information on molecular geometry can be used as an initial guess of the geometry for quantum chemical calculations, while unique identification of bonds and atoms is used for the identification of geometric changes between calculations. For many organics, the geometric information can be automatically generated by translation from InChI or SMILES identifiers using OpenBabel^55^ and by preoptimization using force fields. However, the latter approach is not always suitable for inorganics and thus, storing a precurated geometry can be an advantage.

### 5.2 Navigating Reaction Complexity

**Figure 7:**
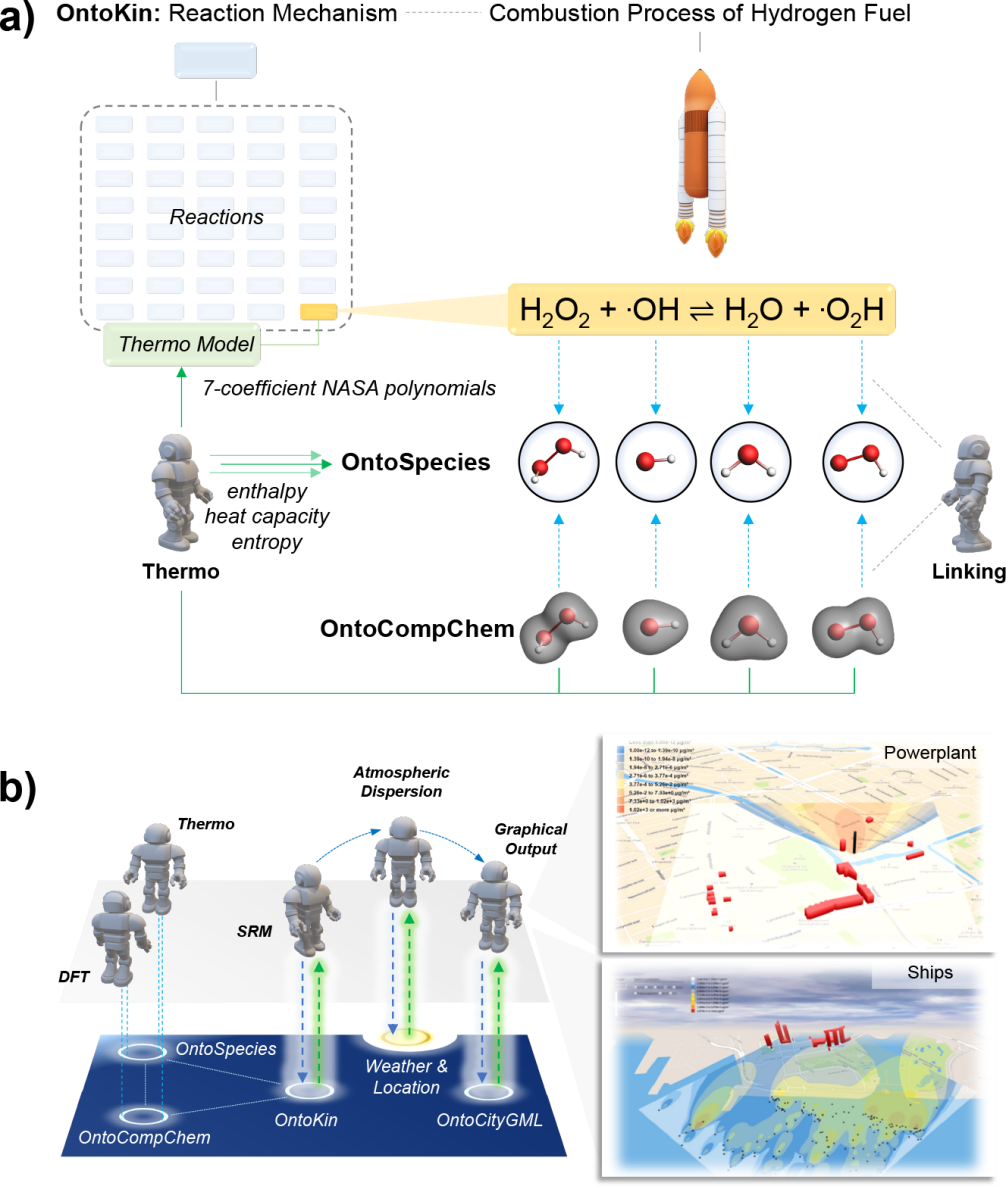
(a) Automated linking between OntoSpecies, OntoKin, and OntoCompChem. (b) Multiscale modeling of pollution starting with fuel molecules stored in OntoKin. Geospatial images adapted from refs (2 and 59). Copyright 2020 American Chemical Society.

In chemistry, many reactions and self-assembly processes starting with simple molecular precursors often lead to a rich variety of chemical species and (metastable) intermediates. The speciation of molecular metal oxides in solution^56^ or the formation of nanoparticulate carbonaceous materials^57^ are examples of such chemistries. Understanding and modeling these chemistries require a grasp of kinetic and thermodynamic factors. Motivated to model these factors semantically on chemical species, our group developed and interlinked the OntoKin^3^ and OntoCompChem^58^ ontologies (see Figure 7a).

OntoKin is an ontology that represents reaction mechanisms in alignment with nomenclature standards used in computer-aided process design.^3^ In a chemical process, a reaction mechanism constitutes a set of stoichiometric reactions involving different chemical species. In OntoKin, a reaction is described through products and reactants that are further described through different thermodynamic and transport model concepts and identified via OntoSpecies IRIs. Depending on where the reaction occurs, OntoKin introduces further specifications (e.g., in gas, on the surface, etc.). The reaction rate of each reaction is presented based on modified Arrhenius-type rate models, meaning that they allow a variety of temperature and pressure dependencies needed to cover gas-phase kinetics (i.e., computation of rate coefficients). As a single reaction mechanism may consist of many different reactions, OntoKin, in conjunction with OntoSpecies, can provide a facile and unambiguous comparison between other kinetic, thermodynamic, or transport models reported in the literature.^59^ The ontological framework also allows curation of values that experts evaluate as reliable based on explicit mark-up. The OntoCompChem ontology represents the input and output of density functional theory (DFT), currently mainly focused on molecular systems.^58^ OntoCompChem has been developed based on the semantic concepts specified in the CompChem convention of Chemical Markup Language (CML).^60^ A calculation in OntoCompChem is described in terms of (a) its objective (e.g., single point calculation, geometry optimization, or a frequency calculation); (b) the software it uses (e.g., Gaussian16); (c) the employed theoretical level in terms of functional and basis set (e.g., B3LYP, 6-31G(d)); and (d) the overall charge and spin polarization. The ontology also represents the calculated frontier orbitals and the final converged self-consistent field (SCF) energy. For geometry optimizations, the final optimized geometry is represented, while for frequency calculations, it stores the zero-point energy correction and a full list of the computed vibrational frequencies linking back to the stationary geometry and calculation it corresponds to.

A linking agent automates the creation of links between reactions, species, and DFT calculations.^53^ Such an agent is needed because a reaction mechanism in OntoKin can easily involve thousands of species and tens of thousands of reactions.^59^ The linking allows zooming into a mechanism, its reactions, and involved species. An example may be the combustion of clean hydrogen fuel used as a rocket propellant, which involves 10 species and 40 elementary reactions, one of which is H_2_O_2_ + ^•^OH ⇌ H_2_O + ^•^OOH (see Figure 7a). For existing DFT calculations, a Thermo agent instantiates enthalpy, heat capacity, and entropy factors back to the involved species and 7-coefficient NASA polynomials to the reaction. If experimental data is provided as a concept, reaction mechanisms can be linked to it, and agents wrapping our custom-made software can do sensitivity analysis and calibration, providing a quantitative explanation of experimental phenomena.^61^ Finally, a workflow of agents (see Figure 7b) that perform (i) DFT calculations; (ii) thermodynamic data analysis; (iii) stochastic model calculations predicting particle formation from fuels in engines; and (iv) atmospheric dispersion modeling based on real-time weather data and graphical output based on physical infrastructure are showcased to predict the dispersion of particle pollution in urban areas.^2^ The relevance of such systems is in digital urban planning.

### 5.3 Automating Rational Design of Self-Assembled Materials

**Figure 8:**
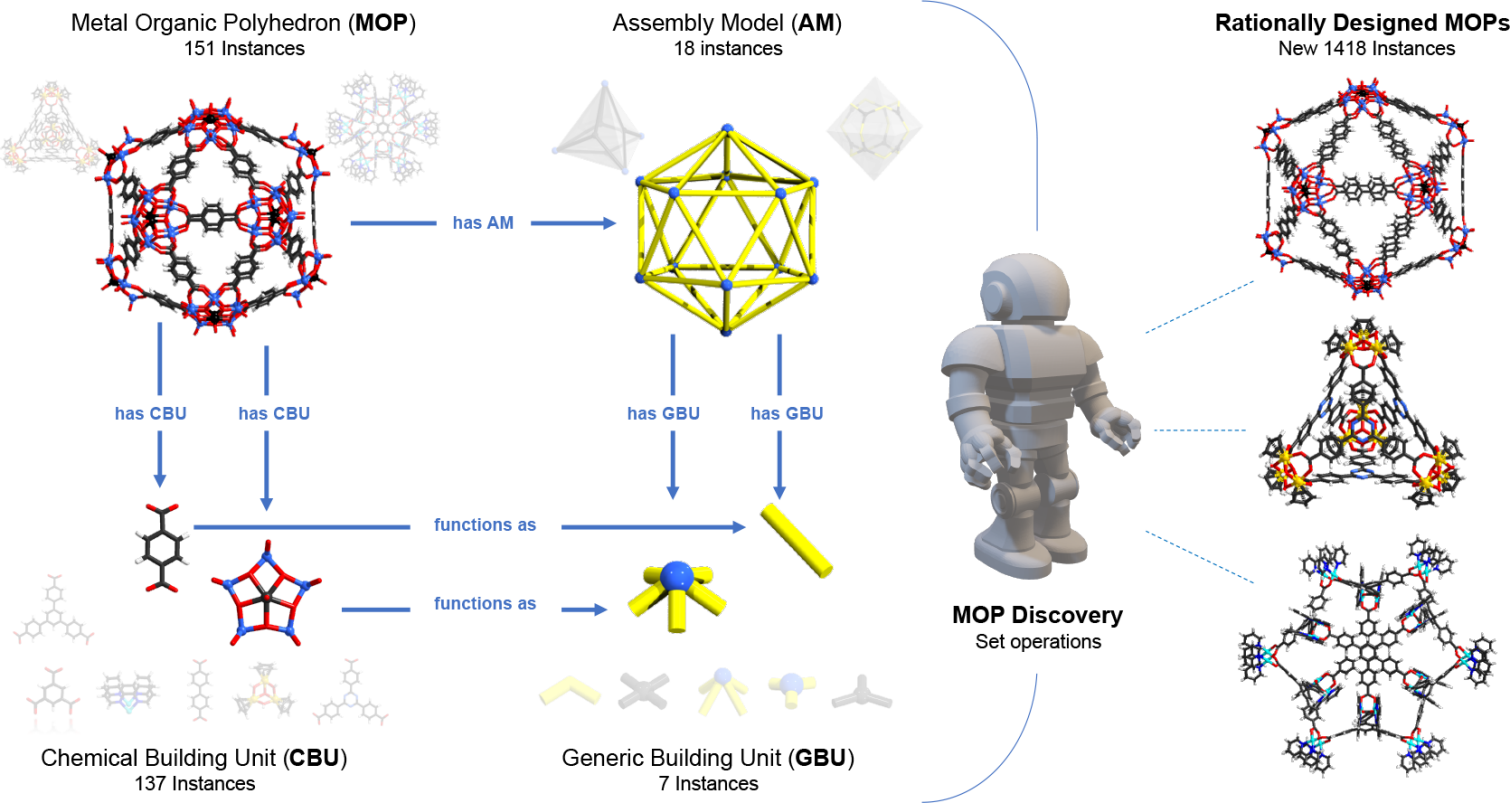
Key concepts in OntoMOPs (left) and examples of newly rationally designed MOPs (right). MOP images adapted with permission from ref (1). Copyright 2022 American Chemical Society.

Metal–organic polyhedra (MOPs) are assemblies made of organic and metal-based chemical building units (CBUs) resembling the shape of regular polyhedra.^62^ MOPs and other cage-like structures are rationally designed by domain experts. To design new MOPs, an expert requires the consideration of both chemical and spatial complementarity factors. Insights from didactical research with toys have shown that children do not need any formal foreknowledge on geometric aspects to build polyhedral models,^63,64^ which implies that some form of mental imagery is involved as part of the overall reasoning. These considerations inspired the conceptualization of assembly models (AMs) and generic building units (GBUs) as mental blueprints involved in the rational design of MOPs from sets of available CBUs (see Figure 8).^1^ The latter concepts were encoded in the OntoMOPs ontology, where the CBUs were further instantiated as species based on the OntoSpecies ontology. The ontology further allows labeling MOPs with their provenance, which in this case was the digital object identifier of the work in which they have been reported. The MOP discovery agent was based on an algorithm that performs set operations revealing which CBUs can be meaningfully combined without causing undesired strains. The study involved 151 experimentally reported MOPs built from 137 unique CBUs, which were effectively clustered in 18 AMs and 7 GBUs, respectively. The MOP discovery agent showed that up to 1418 new MOP instances could be rationally designed, some of which are confirmed by domain experts. The latter aspect is a considerable advantage as it allows more focused and efficient exploration of chemical spaces through calculations and experiments.^1^ The rational projection estimate is a significant reduction in the combinatorial chemical space, which in this case amounts to about 80 000 possibilities.^1^

### 5.4 Marie: Enabling User-Friendly Interaction with TWA KG

**Figure 9:**
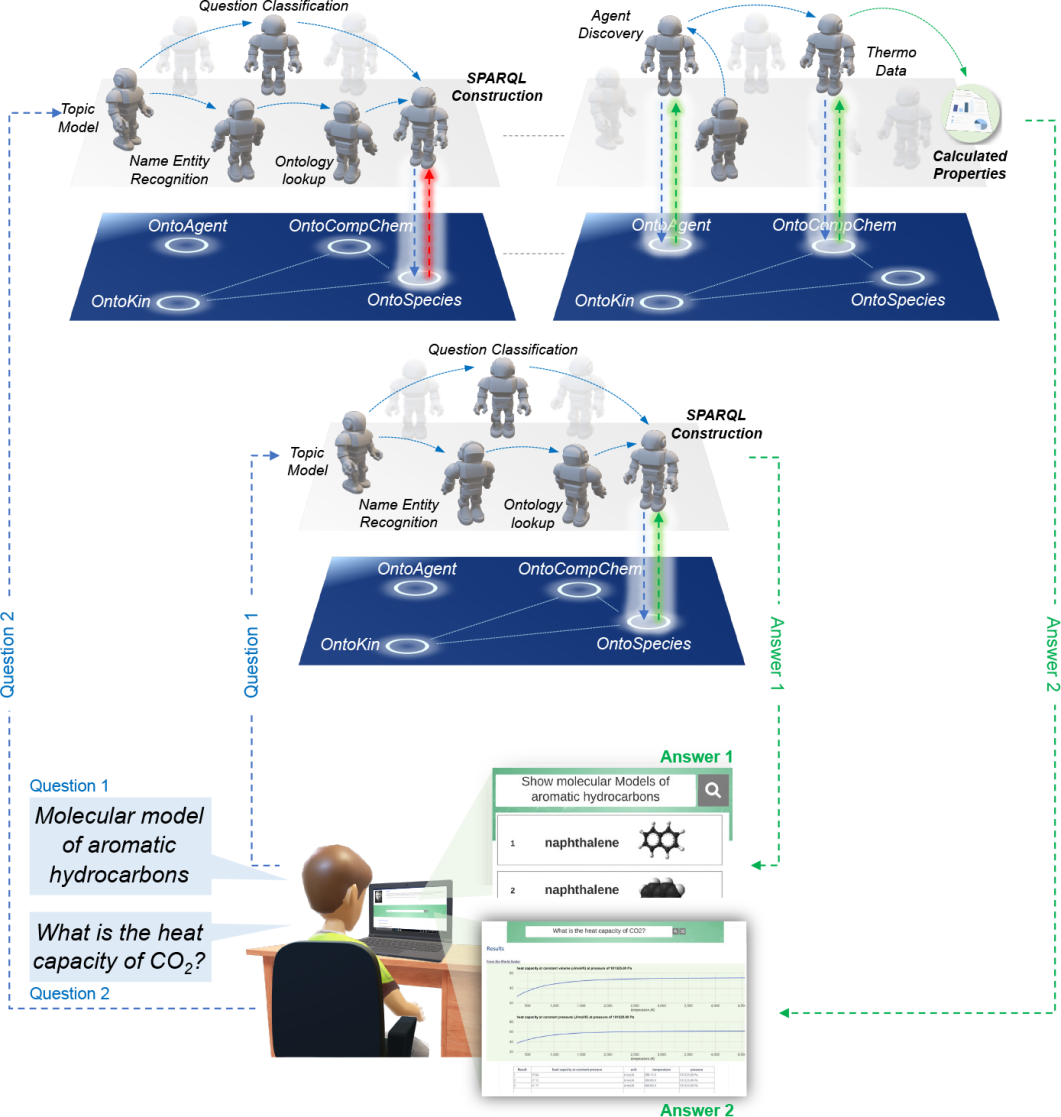
Marie’s back-end operations involved in querying information that is in TWA KG and one that it is generated through agent operation. Printed results of queries are adapted with permission from (i) ref (4), Copyright 2021 American Chemical Society, and (ii) ref (65), Copyright 2022 Elsevier Ltd.

Querying a KG requires the use of a query language (e.g., SPARQL) and awareness of how the knowledge has been structured in that KG. These factors make exploration of the KG less convenient for users who lack the foreknowledge; thus, a more user-friendly interface with the KG is desired. In the context of chemistry within TWA, “Marie” is a question-answering interface that is aimed at allowing users to type their questions in their natural language, which are then translated behind the scenes into machine readable queries.^4,65^ To achieve this, Marie implements natural language processing (NLP) and a network of agents that can identify the topic, the type of question, and the entities the user is asking about. Once clarified, the agents pass the information to an ontology lookup agent that passes the information to a SPARQL construction agent, which then queries the KG and returns information to the user.^4^ A typical example is when a user asks Marie to show models of aromatic hydrocarbons (see Figure 9).^4^

As it is challenging to store all knowledge, while much knowledge can be indirectly inferred or calculated, Marie takes a different circuit when the answer is not found in the KG. In this case, an agent that discovers agents is activated, who then allocates an appropriate agent for the task. The appropriate agent can then query the graph and calculate information. An example would be a question to display the heat capacity of CO_2_, where the Thermo agent can calculate it from instances in OntoCompChem.

## 6 Real-Time Knowledge Dynamics

Many discoveries or outcomes in chemical research depend on other outcomes in the field or, more generally, from the real world. For instance, when a chemist plans the synthesis and the characterization of a new chemical, what instrumental infrastructure will be used is dependent on the nature of the chemical target. Further on, the discovery of new self-assembled material may depend on the discovery of a suitable building block precursor. Navigating dependencies is a complex and challenging task; however, its successful emulation provides an opportunity to realize autonomous laboratory systems,^66^ and even future AI Scientists.^67^

The dynamic data-driven applications systems (DDDAS),^68^ which originated from control systems, is a research paradigm focused on tackling this challenge. It seeks to provide data context to improve decision-making in dynamic and complex environments. Using the KE approach, our group has worked on a derived information framework as a knowledge-graph-native solution to represent how pieces of information depend on others in a dynamic knowledge graph. The framework represents complex and interconnected phenomena as a directed graph of computational or physical activities, with agents serving as executable knowledge components. Once dependencies between objects are created, the framework propagates the effects induced by changes in the source information. We envisage the DDDAS framework providing solutions to the aforementioned difficulties in the chemistry domain. Considering the ontological extensibility of the KG, such frameworks are expected to be compatible with the implementation of strategies that take into consideration the propagation of errors.^69,70^

## 7 Summary and Outlook

In this Account, we have summarized the developments of KE in chemical research. From its beginnings, KE in chemistry has been going through a very challenging path and generally has retained its relevance through the engineering of expert and knowledge management systems.^14^ The beginning of the Semantic Web opened a new paradigm for KE, effectively removing any boundary in terms of knowledge representation and reasoning. By building a KG that includes agents, a new ecosystem for chemical knowledge creation and exploitation has been enabled, allowing the implementation of inductive and, hopefully, over time, abductive reasoning algorithms as well. These aspects have been recently showcased to scale up discoveries^1^ and likely are a path forward to chemical intelligence amplification.

Through multiple examples of our work, we see that a KG with its agents can combine complex decision-making processes with the generation of new knowledge from calculations, external sources, and in the near future, autonomous experiments.^66^ Based on this, it is not difficult to envision more sophisticated combinations of agents involved in the conceptualization and creation of new molecules and materials in near future. Making chemical knowledge part of a single knowledge ecosystem enables efficient inferring across disciplines, scales, and depths in terms of chemical space exploration. In this regard, KE can be a true enabler of systems-level research frontiers such as materiomics and systems chemistry. Much of the success in the latter will be critically dependent on the wisdom of the human experts in structuring knowledge and their capability of developing rational agents. Finally, we expect that the enormous progress in machine learning combined with ideas of KE will further expand the knowledge space.
